# Multi-Method Approach in the Assessment of Alexithymia in Patients With Scleroderma: Use of Two Different Tools

**DOI:** 10.3389/fpsyg.2021.696345

**Published:** 2021-11-29

**Authors:** Anna Dattolo, Tonia Samela, Damiano Abeni, Sabatino Pallotta, Francesca Sampogna

**Affiliations:** ^1^Clinical Epidemiology Unit, IDI-IRCCS, Rome, Italy; ^2^Dermatology Unit, IDI-IRCCS, Rome, Italy

**Keywords:** alexithymia, scleroderma, systemic sclerosis, TAS-20, TSIA

## Abstract

**Objective:** Alexithymia is frequent in patients with some chronic dermatological conditions. The aim of the study was to measure the prevalence of alexithymia in patients with systemic sclerosis (SSc) using two different tools.

**Methods:** Consecutive patients diagnosed with SSc were recruited at day hospital and hospitalization regimen. Alexithymia was measured using the self-administered questionnaire 20-item Toronto Alexithymia scale (TAS-20) and the semi-structured interview 24-item Toronto Structured Interview for Alexithymia (TSIA).

**Results:** The study sample consisted of 67 female patients, aged from 29 to 82 years. According to TAS-20, 22.4% of patients were alexithymic and 17.9% were borderline alexithymic. Also, in our sample mean TAS-20 value was 48.9 and TSIA mean value was 20.3. Spearman’s correlation coefficient between TAS-20 total score and TSIA total score was 0.603. A high correlation was observed between TAS total score and the “Affective Awareness” (AA) scale of the TSIA. TSIA total score significantly correlated with the “Difficulty describing feeling to others” (DDF) scale of the TAS-20. The highest correlation between scales of the two instruments was that between TAS-20 DDF and TSIA AA (*r* = 0.675).

**Conclusion:** The prevalence of alexithymia in SSc patients was higher than in the general population, and similar to that of psoriasis patients. The measurements obtained using the TAS-20 and the TSIA were highly correlated. However, they also showed some diversities in the scales, indicating that they may measure different domains.

## Introduction

The skin, more than any other organ of the human body, expresses our experiences, our distress, our traumas. The skin is a privileged place where the *ex emotione* disease may rise. It is the psychosomatic organ par excellence. Psychiatric and psychological factors play an important role in several dermatological conditions (Gupta and Gupta, 2003). They may act as triggering and exacerbating factors, and they may be a consequence of the heavy impact of the condition on patient’s life. The most studied psychiatric conditions in dermatology are depression and anxiety, and among psychological factors shame, helplessness and anger (Sampogna et al., 2012; Pärna et al., 2015). There is a high risk of comorbidity with psychiatric conditions in dermatological diseases such as psoriasis (Evers et al., 2005; Janković et al., 2009), hidradenitis suppurativa (Misitzis et al., 2020), atopic dermatitis, alopecia areata, vitiligo, and acne (Gupta and Gupta, 1998).

The relationship between psychological distress and skin alterations has always been a topic of great interest for researchers and clinicians. Unlike many other organs in the body, human skin may express a reaction to psychological stressors (Mento et al., 2020). For these reasons, authors like Paus et al. (2006) formulated the theory of “brain-skin connection.” This theory explains that mental, physical, and emotional distress could affect the skin through a bidirectional pattern (Eming et al., 2007). In fact, in stressful conditionshormones relapse pro-inflammation agents (Dhabhar, 2000) and increase inflammation, with a consequent systemic alteration of neuroendocrine and immunological parameters (Pavlovic et al., 2008). At the same time, skin reactions trigger an abnormal immune-mediated response that could worsen the pre-existing skin condition or prevent the skin healing processes (Eming et al., 2007).

Alexithymia is related to emotional self-awareness and to the ability to regulate one’s emotional states. Emotions are primarily physical events, feelings are mostly mental experiences (Adolphs and Andler, 2018). Alexithymia is a deficit in feelings. The construct of alexithymia has been conceptualized as a transdiagnostic clinical dimension, characterized by specific psychopathological aspect. It includes a group of cognitive and affective contents that had been initially observed in patients with “classic” psychosomatic diseases (e.g., peptide ulcer, asthma, ulcerative colitis, rheumatoid arthritis, hypertension) and subsequently also in psychiatric patients with substance use disorder, post-traumatic stress disorder, somatization disorder, and eating disorders (Nemiah and Sifneos, 1970). This type of alexithymia, defined as secondary, has to be distinguished from organic alexithymia, which refers to a condition in which alexithymia is due to an organic damage of the brain structures involved in emotional processing. Secondary alexithymia is instead a consequence of events occurring during life (Taylor et al., 2003). Several studies have shown that alexithymia is multidimensional and includes both emotional and cognitive components. The emotional components are characterized by the incapacity to describe and identify feelings, and the cognitive components are characterized by the deficit of daydreaming and the externally oriented thinking. Beyond the theoretical debate, most assessment tools that evaluate alexithymia measure both components as a single construct. The salient features of the construct of alexithymia are: difficulty in identifying feelings and distinguishing between feelings and physical sensation of emotional activation; difficulty describing emotional feelings to others; reduced imagination and externally oriented cognitive style. These characteristics are linked to deficits in emotions’ processing and regulation (Bagby et al., 2006).

The association between alexithymia and various medical disorders suggests that people with alexithymia seem not to cope effectively with environmental stressors because of an altered cognitive (i.e., lack of emotional awareness), behavioral (i.e., maladaptive coping skills and lack of emotional expression), and physiological (i.e., increased arousal) components (Martin and Pihl, 1985). This altered response to stress might prolong and exacerbate the effect of stressors exposure and dysregulate the immune and somato-visceral response (Martin and Pihl, 1985; Padgett and Glaser, 2003; Porges, 2009).

The presence of alexithymia in dermatological patients is controversial. A high prevalence of alexithymia has been observed in patients with psoriasis (Sampogna et al., 2017), hidradenitis suppurativa (Chiricozzi et al., 2018; Quinto et al., 2021), and atopic dermatitis (Chiricozzi et al., 2020). However, no association has been found between alexithymia and acne (Sunay et al., 2011) or seborrhoeic dermatitis (Cömert et al., 2013). To our knowledge, only one study (Basta et al., 2019) evaluated alexithymia in patients with systemic sclerosis, finding a prevalence of 42%. In that study, alexithymic patients presented increased depressive and anxiety symptoms, sleep disorders, facial image dissatisfaction, disability in activities, and reduced health-related quality of life (Benrud-Larson et al., 2003; Basta et al., 2019).

Systemic Sclerosis (SSc) or scleroderma is a chronic, autoimmune, connective tissue disease which involves the vascular system, the skin, and internal organs. It is a rare condition, with a prevalence rate of around 5/100,000. Women are at much higher risk for SSc than men, with a ratio that ranges from 3.1 to 14:1 (Chifflot et al., 2007). It has a high related morbidity and mortality, with a significant impairment on patient’s quality of life (Joachim and Acorn, 2003; Hudson et al., 2009; Cinar et al., 2012; Kwakkenbos et al., 2015). SSc is also associated with frequent fatigue (Assassi et al., 2011), pain (Schieir et al., 2010), anxiety and depression (Müller et al., 2012), sleeping disorder (Frech et al., 2011), and sexual dysfunction (Malcarne et al., 2007; Oksel and Gündüzoglu, 2014). Both physical and psychological issues may affect social relationships, cause functional problems, alter patient’s self-image and trigger body image dissatisfaction (Suarez-Almazor et al., 2007; Van Lankveld et al., 2007; Basta et al., 2019). Given the importance of emotional regulation processes in psychological wellbeing and assumed that alexithymia could be a risk factor for psychopathology, we aimed to add a contribution on this topic detecting the level of alexithymia in a sample of patients with SSc. There are different tools to assess alexithymia in research and clinical practice (Bagby et al., 2006; Caretti et al., 2011). Each of them has pros and cons. The most used instrument for assessing alexithymia is the 20-item Toronto Alexithymia Scale (TAS-20) (Bagby et al., 1994). It is completed by the patient in a short time, and it does not require a specific training to be administered and scored. However, the self-report nature of this tool may lead to a bias in the evaluation. The TAS-20 may underestimate the presence of alexithymia, because this instrument assumes that people have the capacity that alexithymics are supposed to be lacking (i.e., the mental processing of affective states). Also, the TAS-20 does not include items directly measuring the reduced fantasy activity. To address the limitations of this instrument, Bagby et al. (2006) developed the 24-item Toronto Structured Interview for Alexithymia (TSIA) as an alternative measure. This instrument measures Imaginal Processes (IMP) in addition to the TAS-20 components and has the advantage of being administered by a psychologist/psychiatrist. A limitation of this instrument, however, is that it requires training for administration and coding, and that scoring is made by the interviewer according to the answers given by the individuals, and thus the results may be affected by interviewer bias.

The aims of the present study were: (i) to evaluate the prevalence of alexithymia in a sample of patients with SSc; (ii) to explore differences in alexithymia scores according to sociodemographic features of the sample; (iii) to evaluate the convergent validity of the TAS-20 and the TSIA.

## Materials and Methods

### Study Design and Sample

In this cross-sectional study, consecutive patients with a diagnosis of SSc were recruited in day hospital and hospitalization regimen. The inclusion criteria were: age >18 years, and diagnosis of systemic sclerosis (SSc). The exclusion criteria were: inability to understand Italian language, inability to understand the questions, diagnosis of psychiatric disorders in the last 12 months. Initially 87 patients were contacted, and 19 of them refused to participate in the study. Only one man was among those contacted, but was not recruited in order to preserve the homogeneity of the sample.

The study was conducted in compliance with the recommendation of the latest revision of the Declaration of Helsinki, the Oviedo Convention, and the regulations of Good Clinical Practice (GCP). The Institutional Ethical Committee approved the study protocol (Prot. N 14/CE/2018 6/02/2018). Data were collected between January and June 2019.

### Measure of Alexithymia: Toronto Alexithymia Scale-20

The TAS-20 consists of 20 items, each scored using a five-point Likert scale from 1 (strongly disagree) to 5 (strongly agree). It has three factorial scales: difficulty identifying feelings (DIF/F1); difficulty describing feelings to others (DDF/F2); externally oriented thinking (EOT/F3). The TAS-20 does not include the evaluation of the reduced ability to fantasize and access other imaginary processes. In fact, in the creation of the TAS-20, items to assess imaginary processes were eliminated due to a low correlation with the total score and/or a high correlation with social desirability scores. According to some empirical indications, this dimension is indirectly evaluated by the EOT factor of the TAS-20 (Bagby et al., 2006). The total score can vary from 20 to 100, with higher scores indicating higher levels of alexithymia. Individuals are classified according to the total score as follows: ≤51 non-alexithymic; from 52 to 60 borderline alexithymic; from 61 alexithymic. In the original paper (Bagby et al., 1994), the TAS-20 showed a good internal consistency (Cronbach’ alpha = 0.81 for the total scale, 0.78 for DIF, 0.75 for DDF, and 0.66 for EOT), and a test-retest reliability of 0.77. We used the Italian adaptation of the TAS-20 that demonstrated factorial validity, internal consistency (Cronbach’s alpha 0.82 for the total scale, 0.79 DIF, 0.68 DDF, 0.54 EOT), and high test-retest reliability (0.86 for the full scale and 0.83, 0.79, and 0.81 for DIF, DDF, and EOT, respectively) (Bressi et al., 1996).

### Measure of Alexithymia: Toronto Structured Interview for Alexithymia

The TSIA is a semi-structured interview composed of 24 items. The hierarchic factor structure consists of four subscales (each with six items): difficulty identifying feelings (DIF); difficulty describing feelings (DDF); externally oriented thinking (EOT) and imaginal processes (IMP). These dimensions are grouped into two higher orders: AA, affective awareness (subscales DIF and DDF); OT, operational thinking (subscales EOT and IMP). Each question is scored on a three-point Likert scale (0–2). Items refer either to the frequency or the intensity of a specific characteristic. For each item there is a series of inquiry questions to elicit information that can help to accurately evaluate each dimension. Higher scores indicate a higher degree of alexithymia and total scores range from 0 to 48. Cut-off scores for the TSIA have not been defined by the literature yet.

The TSIA and its six scales demonstrated acceptable levels of interrater internal and test-retest reliability. In the original paper (Bagby et al., 2006) Cronbach’s alpha were: 0.86 for the total scale, 0.70 (DIF), 0.80 (DDF), 0.70 (EOT), and 0.61 (IMP). Test-retest reliability coefficients were: 0.67 (total TSIA); 0.63 (DIF), 0.53 (DDF), 0.65 (EOT), and 0.88 (IMP). We used the Italian version of the TSIA (Caretti et al., 2011), where Cronbach’s alpha were: 0.86 for the total scale, 0.70 (DIF), 0.75 (DDF), 0.70 (EOT), and 0.74 (IMP). The intraclass correlation coefficients for interrater reliability were: 0.94 (total TSIA); 0.93 (DIF), 0.89 (DDF), 0.93 (EOT), and 0.95 (IMP). A double-blind evaluation was performed by two qualified psychologists who were trained to administer TSIA through a specific university training and a >95% accordance was obtained. For discordant evaluations a briefing was organized between the two judges to reach an agreement.

### Study Procedures

Consecutive patients with SSc from the day hospital or the dermatology unit were contacted and informed about the project. Patients who fulfilled the inclusion criteria and accepted to participate signed a consent form and were included in the study. The TAS-20 was left to the patient, while the TSIA was administered by a psychologist. Data were collected on sociodemographic features (e.g., age, marital status, educational level, occupation, patient hospitalized, or in day hospital).

### Statistical Analysis

Categorical variables were described as numbers and frequencies, while continuous variables as means and standard deviation. For aim (ii) chi-square test (for TAS-20 total score), and *t*-test or ANOVA (for each of the three scales of the TAS-20, and for the TSIA and its subscales) were used to compare alexithymia mean scores in different subgroups of patients according to sociodemographic variables. For aim (iii), convergent validity among scales was evaluated using Spearman’s rho (ρ) correlation coefficient. Correlation was also calculated among all TAS and TSIA scales, age, and education. Cronbach’s alpha was calculated to evaluate the internal consistency of the instruments. As to sample size, we hypothesized a prevalence of alexithymia of 25%, on the basis of data from other chronic dermatological diseases, such as psoriasis (24.8%) (Sampogna et al., 2017) and atopic dermatitis (27.7%) (Chiricozzi et al., 2020). In a sample of 60 patients, the 90% confidence interval is within ±8.5% of the measured value.

## Results

Our sample (Table 1) included 67 female patients (participation rate 77.9%), aged from 29 to 82 years. Mean age was 57.2 years with standard deviation of 13.5.

**TABLE 1:** Percentage of patients with alexithymia or borderline alexithymia according to the 20-item Toronto Alexithymia scale (TAS-20).

**Variable**	**Level**	***N* (%)**	***N* (%) TAS-20 > 51**	*p* *
Overall		67 (100.0)	27 (40.3)	

Age (years)	<50	19 (28.3)	5 (26.3)	
	50–59.9	19 (28.3)	9 (47.4)	
	≥60	29 (43.4)	13 (44.8)	0.335

Education	primary/secondary	23 (34.8)	11 (47.8)	
	high school	30 (45.5)	12 (40.0)	
	university	13 (19.7)	3 (23.1)	0.343

Marital status	single	12 (18.5)	3 (25.0)	
	with partner	44 (67.7)	16 (36.4)	
	separated/divorced	3 (4.6)	2 (66.7)	
	widow/er	6 (9.2)	3 (66.7)	0.259

Occupation	employed	32 (47.8)	10 (31.3)	
	housewife	12 (17.9)	2 (16.7)	
	unemployed	3 (4.4)	2 (66.7)	
	retired	16 (23.9)	11 (68.8)	
	disabled	4 (6.0)	2 (50.0)	0.036

Hospitalization	day-hospital	33 (49.3)	12 (36.4)	
	hospitalized	34 (50.7)	15 (44.1)	0.346

*From chi-square test.

According to TAS-20, 22.4% of patients were alexithymic and 17.9% were borderline alexithymic. These two percentages were pooled together, in order to compare different subgroups of patients. Prevalence of alexithymic patients was significantly higher in unemployed, retired, and disabled compared to employed patients. There was also a nearly statistically significant linear trend (*p* = 0.056) showing a higher prevalence of alexithymic in single patients compared to patients with partner and separated/divorced/widow (Table 1).

Concerning the three dimensions of the TAS-20 (Table 2), DIF was significantly higher in patients aged 50 years or higher, separated/divorced or widow, unemployed, and retired or disabled. There was no significant difference for the DDF dimension among subgroups, except for education (higher values for low education). The mean value of the EOT dimension of the TAS-20 was higher in older, less educated, retired, and disabled patients.

**TABLE 2:** Mean values of the three dimensions of the 20-item Toronto Alexithymia Scale (TAS-20) in different subgroups of patients.

**Variable**	**Level**	**DIF**			**DDF**			**EOT**		
		**Mean (sd)**	***F/t***	***p***	**Mean (sd)**	***F/t***	***p***	**Mean (sd)**	***F/t***	***p***
Overall		17.4 (7.0)			12.6 (4.9)			18.9 (4.4)		

Age (years)	<50	13.8 (5.6)			10.6 (4.3)			15.6 (4.5)		
	50–59.9	18.8 (7.4)			13.9 (5.3)			19.9 (4.1)		
	≥60	18.9 (7.0)	3.77	0.028	13.2 (4.7)	2.54	0.087	20.4 (3.4)	9.50	< 0.001

Education	primary/secondary	19.4 (7.4)			14.3 (5.0)			21.0 (4.0)		
	high school	17.1 (6.7)			12.3 (4.7)			18.5 (4.1)		
	university	14.2 (6.2)	2.41	0.095	10.1 (4.0)	3.65	0.034	16.1 (4.4)	6.97	0.004

Marital status	single	14.4 (5.8)			11.3 (4.9)			17.8 (4.6)		
	with partner	16.9 (7.0)			12.4 (4.9)			19.1 (4.8)		
	separated/divorced	21.0 (9.8)			14.9 (5.7)			19.9 (3.2)		
	widow/er	23.8 (5.4)	2.95	0.040	15.1 (4.6)	0.94	0.427	19.0 (2.5)	0.29	0.834

Occupation	employed	16.1 (7.7)			11.8 (4.9)			17.6 (4.7)		
	housewife	14.3 (5.5)			11.7 (5.9)			19.5 (5.0)		
	unemployed	21.0 (2.6)			13.7 (1.5)			15.7 (3.0)		
	retired	21.4 (5.9)			14.5 (4.0)			21.8 (2.5)		
	disabled	19.3 (5.8)	2.66	0.041	14.0 (5.0)	9.06	0.382	20.0 (2.9)	0.29	0.043

Hospitalization	day-hospital	16.7 (6.6)			12.1 (5.5)			19.1 (4.7)		
	hospitalized	18.2 (7.4)	0.78	0.380	13.2 (4.1)	0.98	0.327	18.6 (4.1)	0.21	0.648

*DIF = Difficulty Identify Feelings; DDF = Difficulty Describing Feelings; EOT = Externally Oriented Thinking.*

*sd: standard deviation.*

The mean values of the dimensions of the TSIA are described in Tables 3, 4. Differences among age groups were not significant, however, older patients tended to have higher scores. More educated patients had lower scores in the total score, and DIF, IMP, and OT scales. There were no differences for marital status. Retired patients had higher scores in TSIA total score and DDF (Table 3). Disabled patients had higher scores in TSIA total score (Table 3), IMP, and OT (Table 4).

**TABLE 3:** Mean values of the TSIA total score and sub-dimensions DIF, DDF, and AA in different subgroups of patients.

**Variable**	**Level**	**TSIA tot**			**DIF**			**DDF**			**AA**		
		**Mean (sd)**	***F/t***	***p***	**Mean (sd)**	***F/t***	***p***	**Mean (sd)**	***F/t***	***p***	**Mean (sd)**	***F/t***	***p***
Overall		20.3 (8.1)			3.8 (2.4)			4.1 (3.4)			7.9 (5.2)		

Age (years)	<50	17.1 (7.6)			3.3 (2.1)			3.0 (3.0)			6.3 (4.6)		
	50–59.9	19.8 (9.0)			3.8 (3.0)			3.7 (3.4)			7.5 (5.8)		
	≥60	22.7 (7.2)	2.96	0.059	4.1 (2.2)	0.67	0.514	5.1 (3.5)	2.37	0.101	9.2 (4.9)	1.92	0.154

Education	primary/secondary	23.4 (8.0)			4.0 (2.6)			4.8 (3.7)			8.9 (5.4)		
	high school	19.5 (8.2)			4.2 (2.3)			3.9 (3.3)			8.1 (5.1)		
	university	16.2 (5.8)	3.39	0.026	2.2 (1.9)	3.73	0.029	3.0 (2.5)	1.27	0.287	5.2 (4.1)	2.39	0.100

Marital status	single	18.2 (9.0)			2.8 (2.4)			3.8 (3.6)			6.7 (5.5)		
	with partner	20.0 (8.0)			3.9 (2.6)			3.7 (3.2)			7.5 (5.1)		
	separated/divorced	23.3 (9.4)			5.7 (2.1)			5.7 (3.8)			11.3 (5.8)		
	widow/er	23.0 (7.1)	0.63	0.596	3.7 (1.6)	1.20	0.316	5.8 (3.6)	1.02	0.391	9.5 (4.1)	0.92	0.434

Occupation	employed	17.9 (8.3)			3.4 (2.8)			3.3 (2.9)			6.8 (5.3)		
	housewife	21.5 (6.8)			3.4 (1.2)			4.2 (3.6)			7.7 (4.4)		
	unemployed	13.7 (3.2)			3.7 (0.6)			1.3 (1.5)			5.0 (1.0)		
	retired	24.7 (6.8)			4.6 (2.2)			6.4 (3.4)			11.0 (4.6)		
	disabled	23.0 (9.6)	2.84	0.032	4.5 (3.9)	0.71	0.588	3.0 (3.6)	3.31	0.016	7.5 (7.0)	2.22	0.077

Hospitalization	day-hospital	21.8 (7.8)			3.7 (2.4)			4.4 (3.7)			8.1 (5.5)		
	hospitalized	18.8 (8.2)	2.26	0.138	3.9 (2.5)	0.13	0.721	3.8 (3.1)	0.58	0.451	7.7 (4.9)	0.11	0.745

*DIF = Difficulty Identify Feelings; DDF = Difficulty Describing Feelings; AA = Affective Awareness.*

*sd: standard deviation.*

**TABLE 4:** Mean values of the TSIA total score and sub-dimensions EOT, IMP, and OT in different subgroups of patients.

**Variable**	**Level**	**EOT**			**IMP**			**OT**		
		**Mean (sd)**	***F/t***	***p***	**Mean (sd)**	***F/t***	***p***	**Mean (sd)**	***F/t***	***p***
Overall		5.3 (2.4)			7.0 (2.9)			12.4 (4.3)		

Age (years)	<50	4.7 (2.2)			6.1 (2.8)			10.8 (4.0)		
	50–59.9	5.1 (1.9)			7.2 (2.6)			12.3 (4.1)		
	≥60	5.9 (2.2)	1.74	0.184	7.6 (3.1)	1.50	0.230	13.5 (4.4)	2.38	0.101

Education	primary/secondary	5.7 (2.5)			8.8 (2.5)			14.5 (3.9)		
	high school	5.3 (2.5)			60. (2.8)			11.3 (4.5)		
	university	5.0 (1.9)	0.38	0.688	6.0 (2.2)	8.74	<0.001	11.0 (3.1)	4.94	0.010

Marital status	single	4.3 (2.5)			7.2 (3.4)			11.5 (4.7)		
	with partner	5.5 (2.4)			7.0 (2.8)			12.5 (4.4)		
	separated/divorced	6.3 (2.1)			5.7 (2.1)			12.0 (4.0)		
	widow/er	6.2 (2.1)	1.16	0.331	7.3 (3.1)	0.24	0.868	13.5 (3.3)	0.30	0.823

Occupation	employed	4.8 (2.3)			6.3 (2.9)			11.2 (4.0)		
	housewife	6.2 (2.1)			7.7 (2.3)			13.8 (3.4)		
	unemployed	3.7 (1.2)			5.0 (1.7)			8.7 (2.5)		
	retired	6.1 (2.2)			7.6 (3.0)			13.4 (4.7)		
	disabled	5.2 (4.0)	1.49	0.217	10.2 (1.5)	2.66	0.041	15.5 (4.0)	2.74	0.036

Hospitalization	day-hospital	5.9 (2.2)			7.7 (2.3)			13.7 (3.4)		
	hospitalized	4.8 (2.5)	4.27	0.043	6.4 (3.3)	3.78	0.056	11.2 (4.7)	6.30	0.015

*EOT = Externally Oriented Thinking; IMP = Imaginal Process; OT = Operative Thinking.*

*sd: standard deviation.*

The Spearman’s ρ correlation coefficient between TAS-20 total score and TSIA total score was 0.603 (Table 5), and there was a moderate-high correlation between the DIF (ρ = 0.464) and the DDF (ρ = 0.604) scales of the two instruments. However, no significant correlation was observed between the two EOT scales. The TAS-20 total score significantly correlated with the DIF (ρ = 0.547), DDF (ρ = 0.526), and AA (ρ = 0.625) scales of TSIA. Correlation between TAS and TSIA scores according to age was 0.50 (<50 years), 0.61 (50–59.9 years), and 0.63 (≥60 years). As to education, it was 0.53 for primary/secondary school, 0.50 for high school, and 0.55 for university. In patients with partner correlation was 0.56, and 0.64 in single patients. In employed individuals, correlation between TAS and TSIA was 0.52, in housewives 0.60, in unemployed 0,50, in retired 0.52, and in disabled 0.40. Cronbach’s alpha for the different subscales were: TAS 0.917, DIF_TAS 0.774, DDF_TAS 0.552, EOT_TAS 0.502, TSIA 0.818, DIF_TSIA 0.568, DDF_TSIA 0.779, EOT_TSIA 0.463, IMP 0.713, AA 0.797, OT 0.680.

**TABLE 5:** Spearman’s correlation coefficient among TAS-20 and TSIA total scores, subscales, and socio demographic variables.

	**TAStot**	**DIF_TAS**	**DDF_TAS**	**EOT_TAS**	**TSIAtot**	**DIF_TSIA**	**DDF_TSIA**	**EOT_TSIA**	**IMP**	**AA**	**OT**	**Age**	**Education**
TAStot	1.00												
DIF_TAS	0.91**	1.00											
DDF_TAS	0.86**	0.73**	1.00										
EOT_TAS	0.63**	0.38**	0.37**	1.00									
TSIAtot	0.60**	0.45**	0.63**	0.42**	1.00								
DIF_TSIA	0.55**	0.46**	0.55**	0.27*	0.67**	1.00							
DDF_TSIA	0.53**	0.43**	0.60**	0.24*	0.81**	0.54**	1.00						
EOT_TSIA	0.23	0.15	0.27*	0.21	0.64**	0.29*	0.40**	1.00					
IMP	0.35**	0.22	0.37**	0.31**	0.63**	0.15	0.31**	0.26*	1.00				
AA	0.62**	0.51**	0.67**	0.32**	0.86**	0.82**	0.91**	0.40**	0.29*	1.00			
OT	0.38**	0.24	0.42**	0.38**	0.81**	0.28*	0.45**	0.75**	0.81**	0.44**	1.00		
Age	0.40**	0.31*	0.23	0.42**	0.32**	0.18	0.26*	0.16	0.19	0.28*	0.25*	1.00	
Education	−0.37**	−0.25*	−0.32**	−0.37**	−0.34**	–0.23	–0.18	–0.14	−0.48**	−0.25*	−0.44**	–0.44	1.00

*TAS = Toronto Alexithymia Scale; TSIA = Toronto Structured Interview for Alexithymia; DIF = Difficulty identifying feelings; DDF = Difficulty describing feeling to others; EOT = Externally oriented thinking; IMP = Imaginal processes; AA = Affective awareness; OT = Operational thinking.*

*p < 0.05; **p < 0.001.

## Discussion

In this study on 67 women with SSc, 22.4% were alexithymic and 17.9% were borderline alexithymic, according to the TAS-20. Such prevalence was lower than the one reported in a previous study on patients with SSc (Basta et al., 2019), i.e., 42 and 13%, respectively. This may be due to the different structure of the samples. In fact, the mean age was lower in our study than in the other, and it has been observed that the prevalence of alexithymia is higher in older patients (Mattila et al., 2006). It has been hypothesized that this may be due to a physiological change in the brain of older people. In fact, it has been observed that higher alexithymia scores correlated with reduced bilateral rostral and right dorsal anterior cingulate cortex (ACC) grey matter volume and reduced right rostral ACC volume, which occur at older age (Paradiso et al., 2008).

When considering only established alexithymia (i.e., TAS-20 score ≥61), the prevalence of 22.4% seen among our SSc patients was quite similar to that of patients with psoriasis (24.8%) (Sampogna et al., 2017) and atopic dermatitis (27.7%) (Chiricozzi et al., 2020), while it was lower than the one reported for patients with hidradenitis suppurativa (37.2%) (Chiricozzi et al., 2018). In the general population of other European countries the prevalence of alexithymia is approximately 10% (Mattila et al., 2006; Franz et al., 2008). To our knowledge, no data are available on the prevalence of alexithymia in the Italian general population. However, mean scores observed in the other populations were very similar to the mean values observed in the general Italian population for the TAS-20 (44.7 ± 11.3, Bressi et al., 1996) and the TSIA (18.4 ± 7.9, Caretti et al., 2011), so that we can speculate that also the prevalence of alexithymia would be similar.

Various hypotheses have been formulated to explain the high prevalence of alexithymia observed in these different dermatological conditions. Given the cross-sectional design of all these studies, it is not possible to draw casual inferences on the possible ethiological role of alexithymia. The association between alexithymia and skin conditions may well be bidirectional. On one side, alexithymia may be an indirect risk factor for the development of an abnormal physiological stress response that may lead to autoimmune disease like SSc, on the other side, it may be linked to the clinical worsening of the abovementioned diseases (Martin and Pihl, 1985).

It has even been suggested that alexithymia might be a condition that patients acquired in order to avoid dealing with unwanted emotions (Panayiotou et al., 2015). This would be confirmed by the observation that low emotional awareness, which is the ability to integrate and differentiate emotions, predicts better response to treatment in psoriasis patients (Consoli et al., 2006). Also, alexithymia may represent a risk for the development and worsening of diseases, since people with this trait have an altered response to stress, and thus they seem not to cope effectively with stressors (Martin and Pihl, 1985). Moreover, the tendency to misattribute bodily signals, which occurs in alexithymic patients, might be another mechanism through which alexithymia worsens clinical conditions (Lumley et al., 1996).

A relevant part of the present study concerned the methodological issue of alexithymia measurement. The TAS-20 is the most used instrument, in fact it requires a short time to be completed (about 10/15 min), and is a self-report measure, which does not require a specific training to be administered. However, the EOT dimension appeared unreliable in many studies, a different factor structure emerges depending on the sample, and criterion validity of the instrument has not been studied (Kooiman et al., 2002). Then in the TAS-20, several questions may be difficult to understand for persons with a low educational level. Moreover, paradoxically, alexithymic patients may not answer accordingly to their real status, since they are not aware of their difficulties in recognizing and expressing emotions. On the other side, a semi-structured interview, such as the TSIA, requires at least 40 min to be completed, and requires specific training for its administration and scoring. The advantage of an interview is the interaction of the patient with a psychologist/psychiatrist, which may provide a more valid assessment, by investigating deeply the psychological status of the patients. On the basis of pros and cons of the instruments, a multimethod measurement, using both self-reported instrument and structured interviews, may be useful to reduce measurement bias (Eid and Diener, 2006). In fact, a recent work by Di Monte et al. (2020) used both TAS-20 and TSIA to assess alexithymia levels in obese patients, supporting the importance of a multimethod assessment in some clinical conditions. Also, the employment of behavioral paradigms (Panasiti et al., 2019) can be particularly useful when assessing the interaction between cognitive and emotional processes in patients lacking emotional awareness or lacking the ability to identify/discriminate specific emotions. These tools may help in reducing measurement biases as well, since the validity of the derived accuracy or reaction time indexes is not impacted by impaired emotional awareness and emotion recognition processes. In our study, the correlation between the total scores of the two instruments was high, confirming a good convergent validity between the two measures. Also, the correlation was high between the two DDF scales, moderate between the two DIF scales, and low between the EOT ones. The low correlation observed in EOT may be determined by the fact that, as said above, this dimension in the TAS-20 includes also imaginary processes, which are evaluated separately in the TSIA. In a study on a group of non-clinical subjects (Montebarocci and Surcinelli, 2018) the correlation between TSIA and TAS-20 was quite similar to that observed in our study (*r* = 0.52). However, in that study the EOT scales of TAS-20 and TSIA had a good correlation. Differences between the two studies may be due to the different samples, one including non-clinical subjects and the other a group of patients with a chronic condition.

On the whole, in our study the TAS and the TSIA evaluations in different subgroups of patients according to sociodemographic variables were satisfactorily concordant. Both instruments pointed out a higher prevalence of alexithymia in older patients and in patients with a low educational level Similarly, it has been reported that in patients with psoriasis low alexithymia scores were associated with higher education and health literacy (Larsen et al., 2020).

Discrepancies between TAS and TSIA scores are thus likely due to the intrinsic structure of the instrument, and not to differences according to sociodemographic variables, as age, education, marital status, or occupation. Further research is necessary to investigate in detail the properties of the two instruments.

The semi-structured interview allowed to bring out the difficulties that patients with SSc encountered in expressing their emotions. They often talked exclusively of their illness, as if all their life was focused on that. Patients often reported a sense of confusion, and sometimes an inability to recognize specific moods, to immediately recognize an emotion, and to differentiate anger, sadness, and anxiety. Patients seemed to be concentrated on concrete reality, and left no space to imagination and fantasy, as highlighted by the IMP dimension. On the items that investigate imaginary processes, there were three answers which were most often given to the question: “do you happen to use your imagination?” or “do you rarely have fantasies?” or “do you happen to find yourself immersed in thinking about characters from novels or films?.” A first answer was “no,” argued with “I have other things to think about”; “I don’t have time for imagination”; “I don’t have time to dream”; a second alternative was “yes,” but the arguments remained concrete, in the sense that by imagination they intended to be able to get better, to be able to hold off the disease, or in any case fantasies about the future, but exclusively concerning the pathology; the third alternative answer was “yes, to escape from reality which is sometimes oppressive,” but the fantasies were trivial.

The high scores obtained for EOT showed that patients’ thoughts reflected a marked attention to details of external events, people and places, rather than to “internal experiences,” including how SSc patients considered their feelings and used them as a guide for their behavior. Another aspect concerning affective awareness was given by the following common responses given by several patients: “I have no difficulty in telling what I feel, but I do not do it,” or “it is not difficult for me to talk about my feelings, but I do not do it because others do not understand,” “I am sociable, but I prefer to be alone.” These patients seem to put in place a defensive style, avoiding relationships and difficult situations, in order to minimize the experience of rejection. The acceptance of SSc may be a long process, which includes acceptance of such a serious pathology, which involves periodic hospitalizations and therefore “interruptions from reality”; acceptance of physical changes and therefore an elaboration of the loss of what one was to make room for what they are with their limits, the acceptance of a different life in which they have to learn to live with the disease; the acceptance that the disease will also change family dynamics.

Our study has several limitations. Its cross-sectional design does not allow to determine the direction of the association between alexithymia and SSc. Then, it has not been possible to include any physiological or clinical indices (such as severity of disease), as well as a control group, that may have broadened the scope of our investigation. As for the limited number of study participants, it should be important to consider that SSc is a rare condition, so that to reach larger sample sizes multi-center studies should be encouraged. Also, we found a low Cronbach’s alpha for the EOT scales of both instruments, however, this was also observed also in previous studies (Taylor et al., 2003; Meganck et al., 2011; Montebarocci and Giovagnoli, 2019), and thus it does not seem to be due to our sample.

Thus, the approach to SSc management should be multidisciplinary. Psychological interventions on these patients may contribute to reduce alexithymia and may improve resilience and tolerance to the disease, inducing healthier coping mechanisms that may also have a positive effect on adherence to treatment, thus favoring a better clinical outcome.

## Data Availability Statement

The raw data supporting the conclusions of this article will be made available by the authors, without undue reservation.

## Ethics Statement

The studies involving human participants were reviewed and approved by Istituto Dermopatico dell’Immacolata, IDI-IRCCS. The patients/participants provided their written informed consent to participate in this study.

## Author Contributions

AD conceived the original idea of the study, collected the data, drafted the manuscript, and contributed to data analysis. TS provided the second set of the evaluations of the TSIA and contributed to drafting and editing of the manuscript. DA designed the study and revised the manuscript. SP collaborated in editing of the final manuscript. FS designed the study, analyzed the data, and revised the final manuscript. All authors read and approved the submitted version of the manuscript.

## Conflict of Interest

The authors declare that the research was conducted in the absence of any commercial or financial relationships that could be construed as a potential conflict of interest.

## Publisher’s Note

All claims expressed in this article are solely those of the authors and do not necessarily represent those of their affiliated organizations, or those of the publisher, the editors and the reviewers. Any product that may be evaluated in this article, or claim that may be made by its manufacturer, is not guaranteed or endorsed by the publisher.

## References

[B1] Adolphs R. Andler D. (2018). Investigating emotions as functional states distinct from feelings. *Emot. Rev.* 10 191–201. 10.1177/1754073918765662 30627213 PMC6322839

[B2] Assassi S. Leyva A. L. Mayes M. D. Sharif R. Nair D. K. Fischbach M. (2011). Predictors of fatigue severity in early systemic sclerosis: a prospective longitudinal study of the GENISOS cohort. *PLoS One* 6:e26061. 10.1371/journal.pone.0026061 22022507 PMC3193535

[B3] Bagby R. M. Parker J. D. A. Taylor G. J. (1994). The twenty-item Toronto Alexithymia scale I. Item selection and cross-validation of the factor structure. *J. Psychosom. Res.* 38 23–32. 10.1016/0022-3999(94)90005-1 8126686

[B4] Bagby R. M. Taylor G. J. Parker J. D. A. Dickens S. E. (2006). The development of the Toronto Structured Interview for Alexithymia: item selection, factor structure, reliability and concurrent validity. *Psychother. Psychosom.* 75 25–39. 10.1159/000089224 16361872

[B5] Basta F. Margiotta D. P. E. Mazzuca C. Batani V. Dolcini G. Moras P. (2019). Factors related to alexithymia in patients with systemic sclerosis: a tight relationship with facial image dissatisfaction. *Rheumatol. Int.* 39 461–467. 10.1007/s00296-018-4214-y 30498976

[B6] Benrud-Larson L. M. White B. Heinberg L. J. Boling C. Reed J. Wigley F. M. (2003). Body image dissatisfaction among women with scleroderma: extent and relationship to psychosocial function. *Health Psychol.* 22 130–139. 10.1037/0278-6133.22.2.130 12683733

[B7] Bressi C. Taylor G. J. Parker J. D. A. Brambilla V. Aguglia E. Allegranti I. (1996). Cross validation of the factor structure of the Twenty-Items Toronto Alexithymia Scale: an Italian multicenter study. *J. Psychosom. Res.* 41 551–559. 10.1016/s0022-3999(96)00228-0 9032718

[B8] Caretti V. Porcelli P. Solano L. Schimmenti A. Bagby R. M. Taylor G. J. (2011). Reliability and validity of the Toronto Structured Interview for Alexithymia in a mixed clinical and nonclinical sample from Italy. *Psychiatry Res.* 187 432–436. 10.1016/j.psychres.2011.02.015 21396720

[B9] Chifflot H. Fautrel B. Sordet C. Chatelus E. Sibilia J. (2007). Incidence and prevalence of systemic sclerosis: a systematic literature review. *Semin. Arthritis Rheum.* 37 223–235. 10.1016/j.semarthrit.2007.05.003 17692364

[B10] Chiricozzi A. Esposito M. Gisondi P. Valenti M. Gori N. Giovanardi G. (2020). Disease Severity Is Associated with Alexithymia in Patients with Atopic Dermatitis. *Dermatology* 236 329–335. 10.1159/000507246 32369808

[B11] Chiricozzi A. Giovanardi G. Caposiena Caro D. R. Iannone M. Garcovich S. Dini V. (2018). Alexithymia affects patients with hidradenitis suppurativa. *Eur. J. Dermatol.* 28 482–487. 10.1684/ejd.2018.3368 30325328

[B12] Cinar F. I. Unver V. Yilmaz S. Cinar M. Yilmaz F. Simsek I. (2012). Living with scleroderma: patients’ perspectives, a phenomenological study. *Rheumatol. Int.* 32, 3573–3579. 10.1007/s00296-011-2230-2 22090008

[B13] Cömert A. Akbaş B. Kılıç E. Z. Akın Ö Gökçe E. Göktuna Z. (2013). Psychiatric comorbidities and alexithymia in patients with seborrheic dermatitis: a questionnaire study in Turkey. *Am. J. Clin. Dermatol.* 14 335–342. 10.1007/s40257-013-0019-7 23609607

[B14] Consoli S. M. Rolhion S. Martin C. Ruel K. Cambazard F. Pellet J. (2006). Low levels of emotional awareness predict a better response to dermatological treatment in patients with psoriasis. *Dermatology* 212 128–136. 10.1159/000090653 16484819

[B15] Dhabhar F. (2000). Acute stress enhances while chronic stress suppresses skin immunity: the role of stress hormones and leukocyte trafficking. *Ann. N. Y. Acad. Sci.* 917 876–893. 10.1111/j.1749-6632.2000.tb05454.x 11268419

[B16] Di Monte C. Renzi A. Paone E. Silecchia G. Solano L. Di Trani M. (2020). Alexithymia and obesity: controversial findings from a multimethod assessment. *Eur. Rev. Med. Pharmacol. Sci.* 24 831–836. 10.26355/eurrev_202001_20066 32016988

[B17] Eid M. Diener E. (2006). “Introduction: the need for multimethod measurement in psychology,” in *Handbook Of Multimethod Measurement In Psychology*, eds Eid M. Diener E. (Washington DC: American Psychological Association), 3–8. 10.1037/11383-001

[B18] Eming S. Krieg T. Davidson J. (2007). Inflammation in wound repair: molecular and cellular mechanisms. *J. Invest. Dermatol.* 127 514–525. 10.1038/sj.jid.5700701 17299434

[B19] Evers A. W. M. Lu Y. Duller P. Van Der Valk P. G. M. Kraaimaat F. W. Van De Kerkhoft P. C. M. (2005). Common burden of chronic skin diseases? Contributors to psychological distress in adults with psoriasis and atopic dermatitis. *Br. J. Dermatol.* 152 1275–1281. 10.1111/j.1365-2133.2005.06565.x 15948993

[B20] Franz M. Popp K. Schaefer R. Sitte W. Schneider C. Hardt J. (2008). Alexithymia in the German general population. *Soc. Psychiatry Psychiatr. Epidemiol.* 43 54–62. 10.1007/s00127-007-0265-1 17934682

[B21] Frech T. Hays R. D. Maranian P. Clements P. J. Furst D. E. Khanna D. (2011). Prevalence and correlates of sleep disturbance in systemic sclerosis-results from the UCLA scleroderma quality of life study. *Rheumatology* 50 1280–1287. 10.1093/rheumatology/ker020 21324979 PMC3116211

[B22] Gupta M. A. Gupta A. K. (1998). Depression and suicidal ideation in dermatology patients with acne, alopecia areata, atopic dermatitis and psoriasis. *Br. J. Dermatol.* 139 846–850. 10.1046/j.1365-2133.1998.02511.x 9892952

[B23] Gupta M. A. Gupta A. K. (2003). Psychiatric and Psychological Co-Morbidity in Patients with Dermatologic Disorders: epidemiology and Management. *Am. J. Clin. Dermatol.* 4 833–842. 10.2165/00128071-200304120-00003 14640776

[B24] Hudson M. Thombs D. B. Steele R. Panopalis P. Newton E. Baron M. (2009). Canadian research group: health related quality of life in systemic sclerosis: a systematic review. *Arthritis Rheum.* 61 1112–1120. 10.1002/art.24676 19644906

[B25] Janković S. Raznatović M. Marinkovic J. Maksimović N. Janković J. Djikanoviæ B. (2009). Relevance of psychosomatic factors in psoriasis: a case-control study. *Acta Derm. Venereol.* 89 364–368. 10.2340/00015555-0669 19688147

[B26] Joachim G. Acorn S. (2003). Life with a rare chronic disease: the scleroderma experience. *J. Adv. Nurs.* 42 598–606. 10.1046/j.1365-2648.2003.02663.x 12787233

[B27] Kooiman C. G. Spinhoven P. Trijsburg R. W. (2002). The assessment of alexithymia: a critical review of the literature and a psychometric study of the Toronto Alexithymia Scale-20. *J. Psychosom. Res.* 53 1083–1090. 10.1016/S0022-3999(02)00348-3 12479990

[B28] Kwakkenbos L. Delisle V. C. Fox R. S. Gholizadeh S. Jewett L. R. Levis B. (2015). Psychosocial Aspects of Scleroderma. *Rheum. Dis. Clin. North Am.* 41 519–528. 10.1016/j.rdc.2015.04.010 26210133

[B29] Larsen M. H. Staalesen Strumse Y. A. Borge C. R. Andersen M. H. Wahl A. K. (2020). Relevant associations between alexithymia and health-literacy in persons with psoriasis. *J. Dermatolog. Treat.* 27 1–9. 10.1080/09546634.2020.1756204 32286098

[B30] Lumley M. A. Stettner L. Wehmer F. (1996). How are alexithymia and physical illness linked? A review and critique of pathways. *J. Psychosom. Res.* 41 505–518. 10.1016/S0022-3999(96)00222-X 9032714

[B31] Malcarne V. L. Hansdottir I. McKinney A. Upchurch R. Greenbergs H. L. Henstorf G. H. (2007). Medical signs and symptoms associated with disability, pain, and psychosocial adjustment in systemic sclerosis. *J. Rheumatol.* 34 359–367. 17304659

[B32] Martin J. B. Pihl R. O. (1985). The stress-alexithymia hypothesis: theoretical and empirical considerations. *Psychother. Psychosom.* 43 169–176. 10.1159/000287876 4034888

[B33] Mattila A. K. Salminen J. K. Nummi T. Joukamaa M. (2006). Age is strongly associated with alexithymia in the general population. *J. Psychosom. Res.* 61 629–635. 10.1016/j.jpsychores.2006.04.013 17084140

[B34] Meganck R. Inslegers R. Vanheule S. Desmet M. (2011). The convergence of alexithymia measures. *Psychol. Belg.* 237 237–250. 10.5334/pb-51-3-4-237

[B35] Mento C. Rizzo A. Muscatello M. R. A. Zoccali R. A. Bruno A. (2020). Negative Emotions in Skin Disorders: a Systematic Review. *Int. J. Psychol. Res.* 13 71–78. 10.21500/20112084.4078 32952965 PMC7498125

[B36] Misitzis A. Goldust M. Jafferany M. Lotti T. (2020). Psychiatric comorbidities in patients with hidradenitis suppurativa. *Dermatol. Ther.* 33:e13541. 10.1111/dth.13541 32385861

[B37] Montebarocci O. Giovagnoli S. (2019). Alexithymia, Depression, Trait-anxiety and Their Relation to Self-reported Retrospective Dream Experience. *Am. J. Appl. Psychol.* 8 121–132. 10.11648/j.ajap.20190806.13

[B38] Montebarocci O. Surcinelli P. (2018). Correlations between TSIA and TAS-20 and their relation to self-reported negative affect: a study using a multi-method approach in the assessment of alexithymia in a nonclinical sample from Italy. *Psychiatry Res.* 270 187–193. 10.1016/j.psychres.2018.09.036 30261408

[B39] Müller H. Rehberger P. Günther C. Schmitt J. (2012). Determinants of disability, quality of life and depression in dermatological patients with systemic scleroderma. *Br. J. Dermatol.* 166 343–353. 10.1111/j.1365-2133.2011.10624.x 21916888

[B40] Nemiah J. C. Sifneos P. E. (1970). Psychosomatic illness: a problem in communication. *Psychother. Psychosom.* 18 154–160. 10.1159/000286074 5520658

[B41] Oksel E. Gündüzoglu N. Ç (2014). Investigation of life experiences of women with scleroderma. *Sex. Disabil.* 32 15–21. 10.1007/s11195-013-9334-4

[B42] Padgett D. A. Glaser R. (2003). How stress influences the immune response. *Trends Immunol.* 24 444–448. 10.1016/s1471-4906(03)00173-x 12909458

[B43] Panasiti M. S. Ponsi G. Monachesi B. Lorenzini L. Panasiti V. Aglioti S. M. (2019). Cognitive load and emotional processing in psoriasis: a thermal imaging study. *Exp. Brain Res.* 237 211–222. 10.1007/s00221-018-5416-y 30374785

[B44] Panayiotou G. Leonidou C. Constantinou E. Hart J. Rinehart K. L. Sy J. T. (2015). Do alexithymic individuals avoid their feelings? Experiential avoidance mediates the association between alexithymia, psychosomatic, and depressive symptoms in a community and a clinical sample. *Compr. Psychiatry* 56 206–216. 10.1016/j.comppsych.2014.09.006 25263517

[B45] Paradiso S. Vaidya J. G. McCormick L. M. Jones A. Robinson R. G. (2008). Aging and alexithymia: association with reduced right rostral cingulate volume. *Am. J. Geriatr. Psychiatry* 16 760–769. 10.1097/JGP.0b013e31817e73b0 18697882 PMC2925448

[B46] Pärna E. Aluoja A. Kingo K. (2015). Quality of life and emotional state in chronic skin disease. *Acta Derm. Venereol.* 95 312–316. 10.2340/00015555-1920 24978135

[B47] Paus R. Theoharides T. Arck P. (2006). Neuroimmunoendocrine circuitry of the ‘brain-skin connection. *Trends Immunol.* 27 32–39. 10.1016/j.it.2005.10.002 16269267

[B48] Pavlovic S. Daniltchenko M. Tobin D. Hagen E. Hunt S. Klapp B. (2008). Further exploring the brain-skin connection: stress worsens dermatitis via substance P-dependent neurogenic inflammation in mice. *J. Invest. Dermatol.* 128 434–446. 10.1038/sj.jid.5701079 17914449

[B49] Porges S. W. (2009). The polyvagal theory: new insights into adaptive reactions of the autonomic nervous system. *Cleve. Clin. J. Med.* 76 S86–S90. 10.3949/ccjm.76.s2.17 19376991 PMC3108032

[B50] Quinto R. M. Sampogna F. Fania L. Ciccone D. Fusari R. Mastroeni S. (2021). Alexithymia, Psychological Distress, and Social Impairment in Patients with Hidradenitis Suppurativa. *Dermatology* 237 103–110. 10.1159/000503319 31743903

[B51] Sampogna F. Puig L. Spuls P. Girolomoni G. Radtke M. A. Kirby B. (2017). Prevalence of alexithymia in patients with psoriasis and its association with disease burden: a multicentre observational study. *Br. J. Dermatol.* 176 1195–1203. 10.1111/bjd.15243 27995617

[B52] Sampogna F. Tabolli S. Abeni D. (2012). Living with psoriasis: prevalence of shame, anger, worry, and problems in daily activities and social life. *Acta Derm. Venereol.* 92 299–303. 10.2340/00015555-1273 22678565

[B53] Schieir O. Thombs B. D. Hudson M. Boivin J. F. Steele R. Bernatsky S. (2010). Prevalence, severity, and clinical correlates of pain in patients with systemic sclerosis. *Arthritis Care Res.* 62 409–417. 10.1002/acr.20108 20391488

[B54] Suarez-Almazor M. E. Kallen M. A. Roundtree A. K. Mayes M. (2007). Disease and symptom burden in systemic sclerosis: a patient perspective. *J. Rheumatol.* 34 1718–1726. 17611983

[B55] Sunay D. Baykir M. Ateş G. Ekşioğlu M. (2011). Alexithymia and acne vulgaris: a case control study. *Psychiatry Investig.* 8 327–333. 10.4306/pi.2011.8.4.327 22216042 PMC3246140

[B56] Taylor G. J. Bagby R. M. Parker J. D. (2003). The 20-Item Toronto Alexithymia Scale. IV. Reliability and factorial validity in different languages and cultures. *J. Psychosom. Res.* 55 277–283. 10.1016/s0022-3999(02)00601-3 12932803

[B57] Van Lankveld W. G. J. M. Vonk M. C. Teunissen H. Van den Hoogen F. H. J. (2007). Appearance self-esteem in systemic sclerosis—subjective experience of skin deformity and its relationship with physician-assessed skin involvement, disease status and psychological variables. *Rheumatology* 46 872–876. 10.1093/rheumatology/kem008 17308314

